# Body size trends in response to climate and urbanization in the widespread North American deer mouse, *Peromyscus maniculatus*

**DOI:** 10.1038/s41598-020-65755-x

**Published:** 2020-06-01

**Authors:** Robert Guralnick, Maggie M. Hantak, Daijiang Li, Bryan S. McLean

**Affiliations:** 1 Florida Museum of Natural History, University of Florida, Gainesville, FL 32611 USA; 2 Department of Biology, University of North Carolina Greensboro, Greensboro, NC 27402 USA

**Keywords:** Climate-change ecology, Evolutionary ecology

## Abstract

Body size decline is hypothesized to be a key response to climate warming, including warming driven by urban heat islands. However, urbanization may also generate selective gradients for body size increases in smaller endotherms via habitat fragmentation. Here we utilize a densely sampled, multi-source dataset to examine how climate and urbanization affect body size of *Peromyscus maniculatus* (PEMA), an abundant rodent found across North America. We predicted PEMA would conform to Bergmann’s Rule, e.g. larger individuals in colder climates, spatially and temporally. Hypotheses regarding body size in relation to urbanization are less clear; however, with increased food resources due to greater anthropogenic activity, we expected an increase in PEMA size. Spatial mixed-models showed that PEMA conform to Bergmann’s Rule and that PEMA were shorter in more urbanized areas. With the inclusion of decade in mixed-models, we found PEMA mass, but not length, is decreasing over time irrespective of climate or population density. We also unexpectedly found that, over time, smaller-bodied populations of PEMA are getting larger, while larger-bodied populations are getting smaller. Our work highlights the importance of using dense spatiotemporal datasets, and modeling frameworks that account for bias, to better disentangle broad-scale climatic and urbanization effects on body size.

## Introduction

Body size change has been hypothesized to be a third universal response to climate warming^1^. The rationale for this hypothesis is based on a space-for-time substitution of the well-studied but still-controversial relationship between body size and latitude or temperature in endotherms, e.g. Bergmann’s Rule or cline. While focusing on wing-length relationships across species of birds within a given genus, Bergmann^2^ suggested that heat-loss scales with body size, and this trend should apply over space or time. For hundreds of years, ecogeographers have been assembling further exemplar studies to test “Bergmann’s Rule”, but recent work has cast substantial doubt on the generality of intraspecific Bergmann’s relationships for endotherms^3^. Further, there has been limited empirical support for manifestation of Bergmann’s Rule over time rather than space, especially in mammals^4^, making the generality of body size change in response to modern climate warming difficult to assess.

Much less well studied is how human disturbance and land use change, specifically in the form of urbanization, might be impacting organismal body size as a result of urban temperature changes and the alteration of habitats and food resources. The human-mediated environment can serve as a strong selective pressure given that built environments and associated infrastructure can fragment natural habitats leading to selective release^5^, introduce or intensify heat-island effects^6^, but also facilitate access to novel food sources^7^. Using predictions from island biogeographic theory, Schmidt and Jensen^8^ argued that adaptation to an increasingly fragmented landscape should result in increases in body size for smaller species and decreases for larger ones (i.e., the “Island Rule”) and suggested that body size responses of mammal faunas to urbanization in Denmark may conform to this rule. However, a more thorough examination by Yom Tov and Yom Tov^9^ did not confirm these results for a subset of species. Because the ecophenotypic outcomes of Bergmann’s Rule and the Island Rule are potentially contrasting for small vertebrates in fragmented landscapes, examining the effects of climate and urbanization proxies across the range of a taxon, while controlling for potential biases, is critical. However, we know of no empirical studies that developed such a framework for use on broad-scale mammalian body size dynamics over time and space.

Addressing questions of the generality of recent body size change, and of the dual importance of urbanization and climate in driving that response, requires dense spatiotemporal sampling to provide replicate observations over multiple areas that have experienced differing amounts of climate change and human impact. The challenge is particularly vexing because long-term and systematic monitoring are often incomplete in places where humans are most impacting the environment, and incidental data (e.g., museum specimens) and other geographically targeted census/survey efforts often have inherent spatiotemporal bias that limit broadest use^10^. Nevertheless, assembly of these disparate data streams is an essential remit for effective monitoring of many different types of species traits in global change contexts^11^. Developing bioinformatic workflows for well-sampled species will help establish best-case scenario approaches to this problem and enable less biased monitoring efforts into the future.

**Figure 1:**
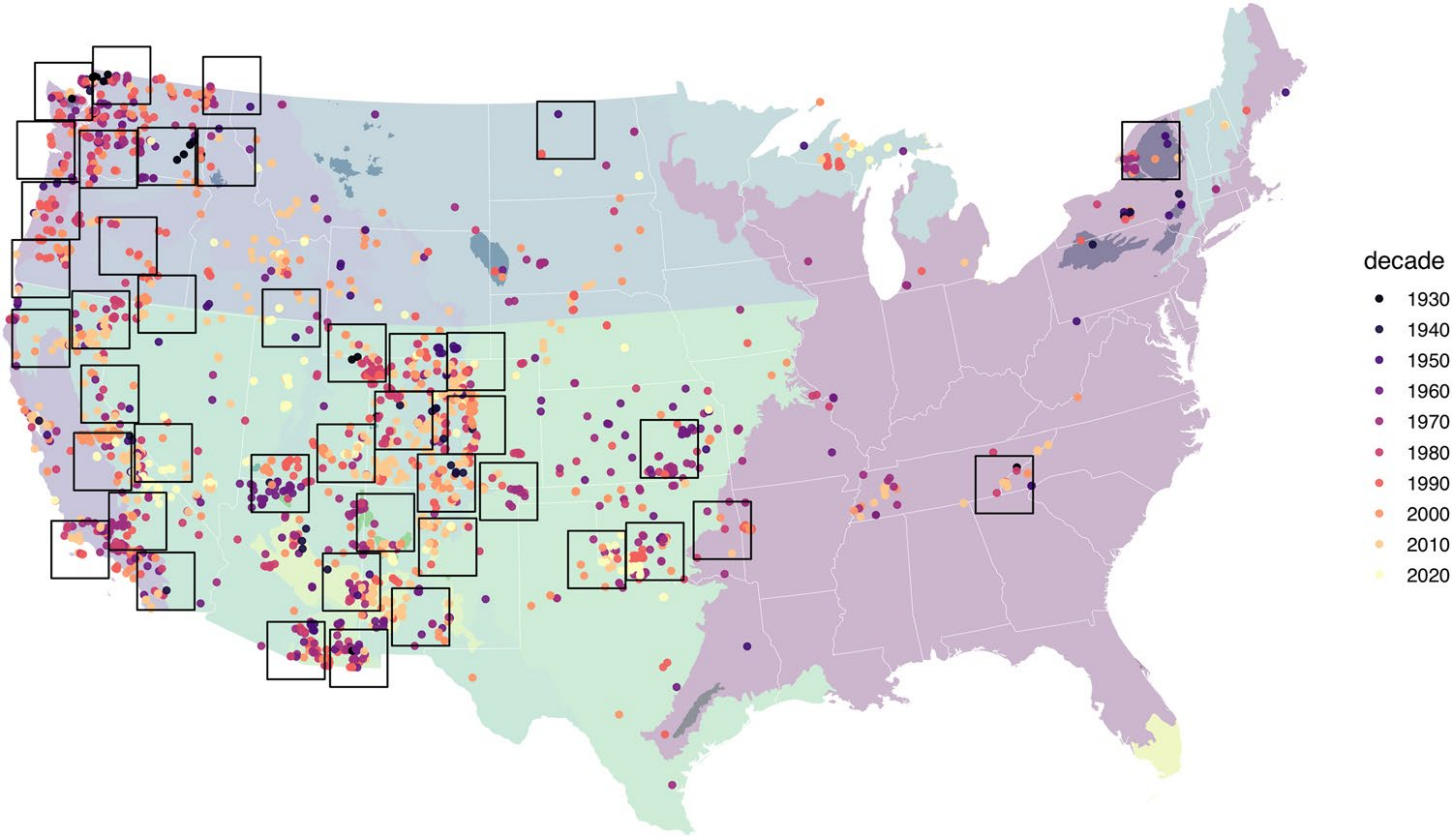
Exemplar zonation using all body mass records from 1930 onwards with records colored by decade (1900–1930 were excluded due to sparse records). Each 200 × 200 km grid cell is a zone. We also color key ecoregions used, shown in the background map of the United States of America. Supplementary Fig. 1 shows the ecoregional designations. This map was created with R version 3.6.2 (https://www.r-project.org/).

*Peromyscus maniculatus* (abbreviated PEMA here), the North American deer mouse, is an ideal species to test these questions since it is widely distributed across North America (Figs. 1 and S1), and without question, is the best sampled rodent in North American museum collections and historical and current census efforts. PEMA have been collected agnostically as part of natural history trapping for hundreds of years^12,13^, resulting in thousands of standard, external morphological measurements on specimen tags and in field notes, many of which have been mobilized to aggregators such as VertNet with collections digitization efforts^14^. In addition, complimentary data sources can also be brought to bear regarding PEMA body size, including historic, continent-wide censuses (e.g.^15–23^), and small mammal trapping data currently being collected by the National Ecological Observatory Network (NEON). Excellent representation across biodiversity data streams makes PEMA an ideal case study for examining spatiotemporal body size trends in the context of global change drivers, while also parsing the biases associated with opportunistic sampling and a diversity of data sources.

Previous studies have found limited support for Bergmann’s Rule intraspecifically in PEMA, as well as interspecifically among other *Peromyscus*. Indeed, the largest *Peromyscus* lineages tend to occur in warmer climates of the subtropics^24–26^. Moreover, ecomorphological variation within and among species - including that potentially associated with thermal biology - tends to be allocated to external (pelage length, tail, ear, hind foot) or skeletal features rather than body size *per se*^25,27^. In a previous analysis, Smith and McGinnis^28^ found no relationship between body size and either latitude or altitude in PEMA and likewise, Wassermann and Nash^29^ found no body size variation along altitudinal transects in Colorado. Hayward^30^ argued that stable microclimates used by PEMA across its range reduce selective pressure on body size as an axis of thermal adaptation. Conversely, a Bergmann’s cline was supported when directly using temperature as a covariate in the closely-related white-footed mouse, *Peromyscus leucopus*^31^.

Our understanding of ecogeographic trends in PEMA has been clouded by use of indirect proxies of temperature (i.e., latitude, altitude), incommensurate body mass metrics (body mass, total length, or head-body length), and studies that often mixed interspecific and intraspecific datasets. This prevents us from identifying whether PEMA body size is a potential axis of phenotypic response to global change. Therefore, in this study, we utilize an approach to spatially and ecologically stratify PEMA records to examine trends in body size in relation to key climate variables and over time. We also focus not only on climatic variables, but on the landscape factor of human population density, which can serve as a broad proxy of urbanization as well as vegetation disturbance^32^. Furthermore, we consider two different body size proxies that may yield different signatures of thermal adaptation: body length and body mass. While many evolutionary studies have advocated using mass (e.g.^33^), as it is a better reflection of overall body size, it can also vary substantially in relation to age, reproductive status (i.e., pregnancy for females, testis development for males), and sex (e.g., sexual dimorphism). Furthermore, PEMA has a generalist diet across much of its range, and seasonal shifts in availability of food resources can be expected to influence body mass of individuals locally^34–36^. Therefore, an additional key question we address is the relationship between head-body length and body mass in PEMA, and how choice of body size proxy can impact our understanding of how species respond to environmental changes.

This work is focused on two main and interrelated questions. The first, purely spatial question is whether climate and human population density, as a proxy for urbanization, impact body size. Our predictions are that PEMA generally follows Bergmann’s Rule when utilizing direct comparisons to temperature, but that fragmentation and availability of anthropogenic food resources for a small, generalist omnivore, such as PEMA, promotes increased body mass and head-body length in urbanized environments. However, it is also possible that heat island effects resulting from urbanization work in opposition to these other factors to reduce body size, leading to no significant changes. The second question focuses on temporal trends and whether PEMA body size has shifted in areas that have experienced increased warming and urbanization. In both of these analyses, we examine body mass and head-body length separately to quantify whether different body size proxies give differing results. Our methodological approach, and attention to bias while using integrated data resources provide not only surprising results about drivers of PEMA body size, but also reproducible methods. We also highlight potential new challenges and opportunities for integrating diverse data streams in monitoring of species traits.

## Results

**Figure 2:**
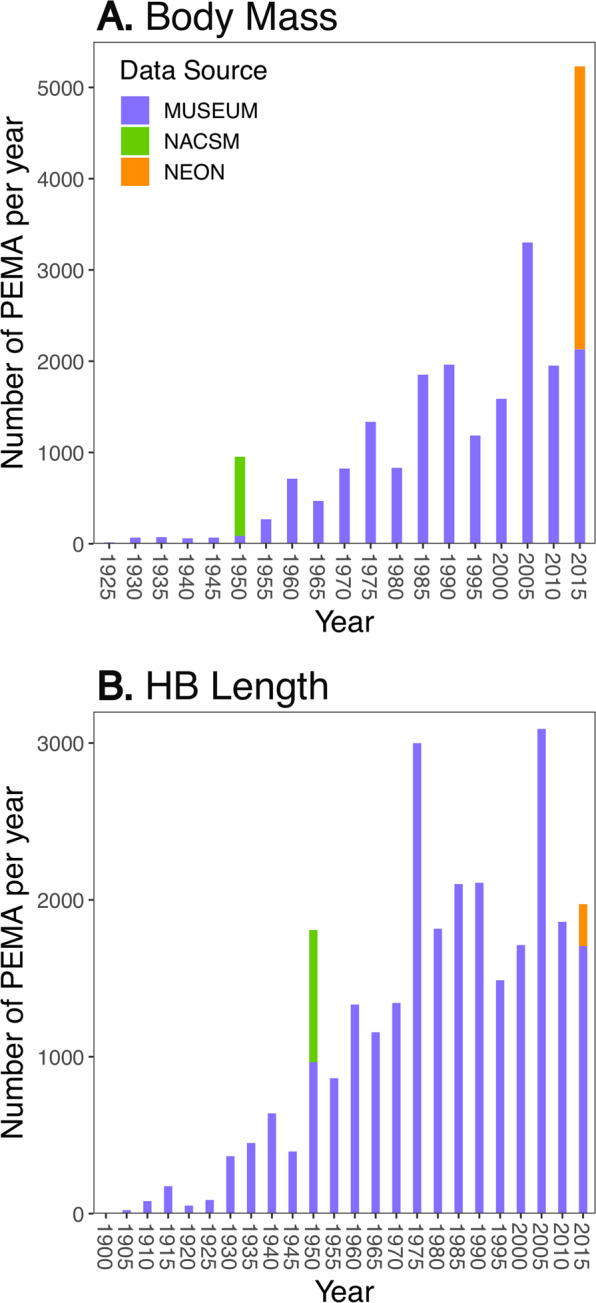
Number of (**A**) body mass or (**B**) HB Length records for PEMA by five year bins from 3 different sources. The vast majority of records come from digitized natural history collections but were supplemented by records from national survey efforts in the late 1940–1950s and in the present.

Compilation across data sources generated a significant dataset of PEMA trait data, and Fig. 2 provides a view of the data densities over time showing how inclusion of NACSM and NEON augments the data available from aggregated natural history collections. We examine key spatial and temporal trends and drivers of body size change based on this unique dataset below (see Table S1 for model descriptions).

### Head-body length and body mass allometry

We found a moderately strong fit in models that examine if log(HB Length) predicts log(body mass) (adj. R^2^ = 0.45; Fig. S2). Given this result, which shows lack of strong collinearity, subsequent analyses were performed separately for HB Length and for body mass.

### Models of spatial variation

The top models of body size variation across space were consistent across each set of analyses, including with and without juvenile PEMA (Tables 1, S2, S3; Figs. 2, S3–S5; and see Supplementary Results). First and foremost, we examined the “source” covariate to determine if there were systematic biases across the different data sources used here. We found limited biases for body mass estimates across datasets. However, we found that NEON HB Length is consistently smaller compared to museum collections datasets and NACSM, an artifact of that particular measurement being taken from live individuals which are difficult to handle and measured in the field. Thus, we decided to exclude NEON as a source for our main results, but do include results with NEON as a source in supplementals (see Table S3; Figs. S4 and S5).

**Table 1:** Highest-ranking spatial (A-B) and temporal (C-D) model predictors for body mass and HB Length relationships. Results are based on datasets without juveniles or NEON as a source. Direction (all negative) of continuous predictor estimates are provided. All predictors in each model were highly significant. The ∆AICc describes the difference in AICc scores between the first and second ranked models.

Top Model	N	*k*	AICc	AICcWt	∆AICc	Marginal R^2^	Conditional R^2^
**(A) Spatial: Body Mass**							
MAT (−) + MAP (−) + Season + Sex	19218	10	108019.6	0.844	3.38	0.047	0.125
**(B) Spatial: HB Length**							
MAT (−) + MAP (−) + Season + Sex + Population Density (−)	27192	11	188940.4	1	18.99	0.036	0.074
**(C) Temporal: Body Mass**							
MAT (−) + MAP (−) + Season + Sex + Decade (−)	12361	12	68968.6	0.712	2.10	0.064	0.204
**(D) Temporal: HB Length**							
MAT (−) + MAP (−) + Season + Sex	26823	11	143907.5	0.711	2.42	0.04	0.143

**Figure 3:**
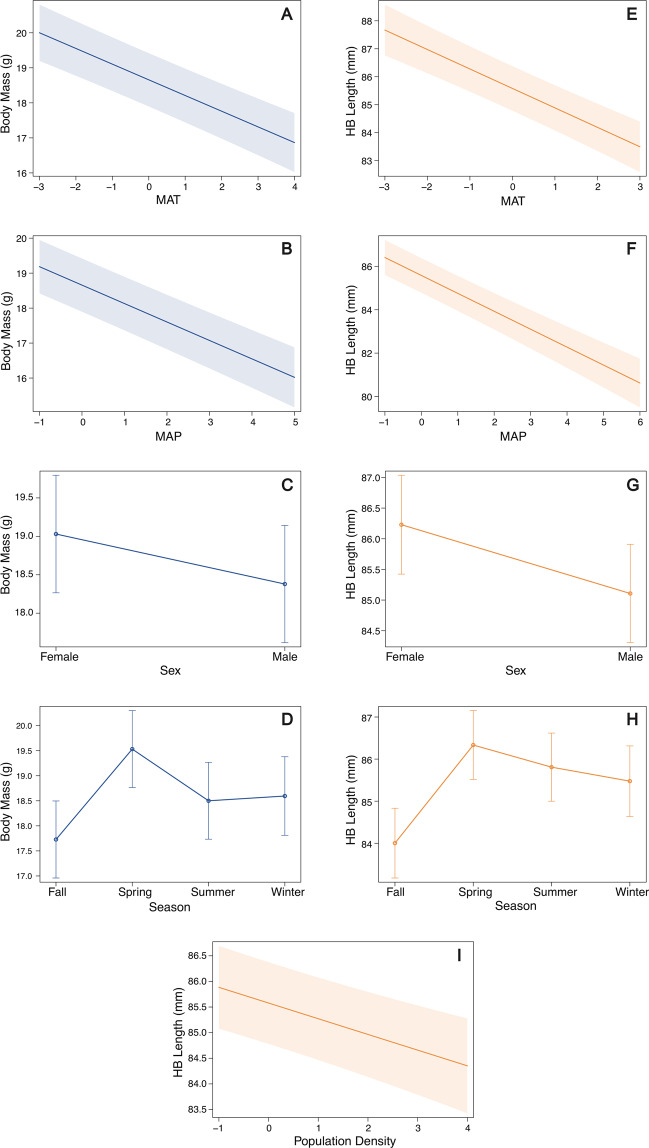
Top spatial model fixed effect plots demonstrating the effects of mean annual temperature (MAT), mean annual precipitation (MAP), sex, season, and population density on PEMA (without juveniles and NEON as a source) body mass (**A**–**D**) and HB Length (**E**–**I**). 95% confidence intervals are included in each plot.

**Figure 4:**
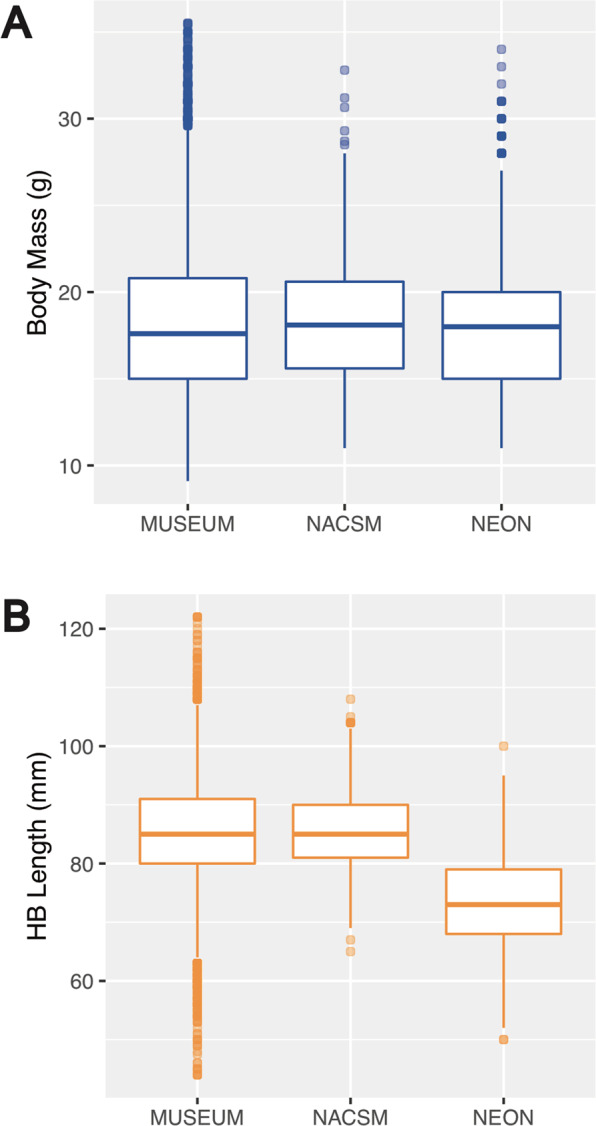
Boxplots of the random effect of source in spatial models for (**A**) body mass and (**B**) HB Length. The thick, horizontal line of each box represents the median body size estimate, boxes indicate the interquartile range, and whiskers extend to the largest and smallest body size estimates.

When examining body mass as a body size metric, we found spatial variation correlated with mean annual temperature (MAT), mean annual precipitation (MAP), season, and sex (Tables 1A, S2a, S3a,c). PEMA body mass is negatively correlated with increasing MAT (*β* = −0.45, *SE* = 0.04, *p* < 0.001; Fig. 3A) and MAP (*β* = −0.53, *SE* = 0.04, *p* < 0.001; Fig. 3B), females are larger than males (*β* = −0.65, *SE* = 0.06, *p* < 0.001; Fig. 3C), and PEMA body mass is lower in the fall compared to other seasons (fall-spring *β* = 1.80, *SE* = 0.10, *p* < 0.001; fall-summer *β* = 0.77, *SE* = 0.09, *p* < 0.001; fall-winter *β* = 0.87, *SE* = 0.13, *p* < 0.001; Fig. 3D). Body mass was variable across source type (variance = 0.04, SD = 0.20; Figs. 4 and S6) and ecoregion (variance = 1.51, SD = 1.23; Fig. S7). When using HB Length as a body size metric, patterns of spatial variation are likewise driven by a combination of MAT, MAP, season, and sex, but also population density (Table 1B, S2b, S3b,d). Like body mass, HB Length is negatively associated with MAT (*β* = −0.70, *SE* = 0.07, *p* < 0.001; Fig. 3E) and MAP (*β* = −0.83, *SE* = 0.07, *p* < 0.001; Fig. 3F). Females display longer HB Length than males (*β* = −1.12, *SE* = 0.10, *p* < 0.001; Fig. 3G). PEMA HB Length is shorter in the fall compared to other seasons (fall-spring *β* = 2.33, *SE* = 0.17, *p* < 0.001; fall-summer *β* = 1.80, *SE* = 0.16, *p* < 0.001; fall-winter *β* = 1.46, *SE* = 0.19, *p* < 0.001; Fig. 3H). Human population density was not an important factor influencing variation of PEMA body mass across the range, but PEMA HB Length decreased with increasing population density (*β* = −0.31, *SE* = 0.06, *p* < 0.001; Fig. 3I). HB Length also varied by source type (variance = 0.77, SD = 0.88; Figs. 4 and S6) and across ecoregions (variance = 1.93, SD = 1.39; Fig. S7).

### Models including decadal covariates

Models of PEMA body mass showed the same trends with and without juveniles (Tables 1C, S2c). Model results with juveniles are included in the supplementary material (Table S2c,d, Supplementary Results). As in models that did not include decadal fixed and random effects, we found that PEMA body mass decreases with MAT (*β* = −0.46, *SE* = 0.07, *p* < 0.001) and MAP (*β* = −0.80, *SE* = 0.06, *p* < 0.001) and females display a larger body mass relative to males (*β* = −0.58, *SE* = 0.07, *p* < 0.001). Further, PEMA consistently have a smaller body mass in the fall season (fall-spring *β* = 2.09, *SE* = 0.14, *p* < 0.001; fall-summer *β* = 0.82, *SE* = 0.13, *p* < 0.001; fall-winter *β* = 1.31, *SE* = 0.17, *p* < 0.001). A key result is that even after accounting for these spatial, climate, and seasonal covariates, we found that PEMA body mass has, overall, decreased over time (*β* = −0.23, *SE* = 0.08, *p* = 0.008 for decade covariate). HB Length trends of PEMA were influenced by a combination of MAT, MAT, sex, and season, excluding juveniles (Table 1D), while decade was also included in the most supported model when including juveniles (Table S2d). In models including decadal covariates, PEMA HB Length decreases with increasing MAT (*β* = −0.76, *SE* = 0.10, *p* < 0.001) and MAP (*β* = −1.24, *SE* = 0.10, *p* < 0.001). PEMA are shorter in the fall compared to other seasons (fall-spring *β* = 1.72, *SE* = 0.20, *p* < 0.001; fall-summer *β* = 1.35, *SE* = 0.19, *p* < 0.001; fall-winter *β* = 1.66, *SE* = 0.22, *p* < 0.001), and females are longer (*β* = −1.17, *SE* = 0.11, *p* < 0.001) than males.

**Figure 5:**
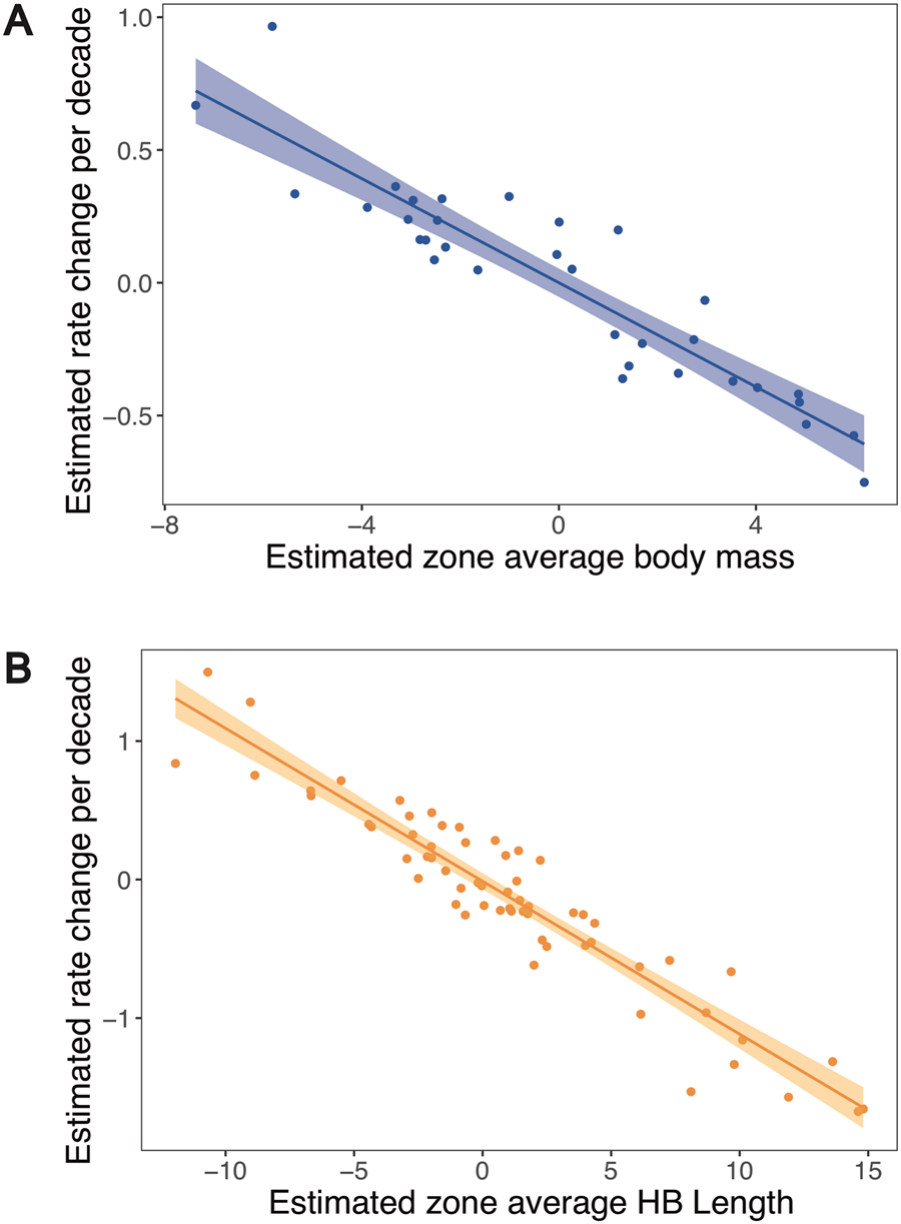
Scatter plot of the random intercepts of zone-averaged PEMA (**A**) body mass and (**B**) HB Length against the random slope of decade (i.e., modeled decadal trait change) from the top-ranked temporal models.

A key finding relates to the random effects. We found there was a strong negative correlation (r^2^ = −0.93) between the random slope (decade) and intercept (spatial zone) for PEMA body mass, indicating that over time, heavier PEMA populations have experienced reductions in mass, while thinner PEMA populations have gained mass (Figs. 5A, and S8a, S9a). Likewise, there was a strong negative correlation (r^2^ = −0.95) between the random slope (decade) and intercept (zone) for PEMA HB Length, indicating that, across all spatial zones, longer PEMA populations (e.g., those with largest HB Length) are getting shorter over time and shorter PEMA populations are getting longer through time (Figs. 5B and S8b, S9a). To provide another perspective on these trends, we plotted the random slopes for both measures of body size (Fig. S9 for body mass and HB Length). Finally, in order to more fully explore these data, we ran an exploratory analysis of trends in body mass change over time for each zone independently rather than in a single modeling framework, and as expected found overall trends towards decreasing size, excepting those zones with initially the smallest individuals, which instead increased in size.

## Discussion

Much has been made about the potential for global change to impact key functional traits of organisms, in particular body size change in the face of changing climates^1^. Much less well understood is how urbanization - a proxy for various global change drivers such as habitat loss and spread of invasive species - may also impact species traits, partly because these changes likely impose a variety of direct and indirect selection pressures. While there have been local-scale studies identifying the phenomenon of temporal change in body size, such as work on the white-footed mouse, *Peromyscus leucopus*, in Chicago^37^, the challenge has been understanding impacts at broader spatial and temporal extents and identifying drivers of phenotypic shifts. Here we utilized new data resources to examine the relative importance of climate and urbanization as key drivers of body size in the most common rodent in North America, *Peromyscus maniculatus*. We do so by first only considering spatial patterns, utilizing ecogeographic theory to inform our predictions and providing a data-rich test of Bergmann’s rule within this species. We then examine temporal changes in PEMA body size over the last 70 years in order to determine if consistent patterns and drivers emerge across both space and time. We expected a consistent, Bergmann’s-like relationship between temperature and both metrics of body size both spatially and temporally. Expectations of body size change due to urbanization were less clear, with potential decreases in size due to urban heat island effects, but possible compensation due to fragmentation and increased resource availability. Our results surprisingly show that temperature-body size relationships are complex over space and time, and HB Length and body mass are affected by different drivers. We also show an unexpected pattern whereby populations of smaller-bodied PEMA have tended to get bigger and vice versa, but without conclusive evidence for body size homogenization over time.

### Spatial drivers of body size for PEMA

Across the range of PEMA, individuals in colder regions were larger in both body mass and HB Length, a finding that is consistent with Bergmann’s Rule^2^. The generality of Bergmann’s Rule has been called into question^3,38,39^ and conflicting results have also been found as to whether the rule applies better to small versus large mammals^40,41^. Our finding that PEMA conforms to the spatial version of Bergmann’s Rule contrasts with a historic study by Smith and McGinnis^28^, which found no association between body size and either latitude or altitude in PEMA across its range. However, that paper used population averages drawn from the literature and did not consider temperature as a potential driver. Wassermann and Nash^29^ also found no relationship between body mass and altitude; however, this study took place in 5 populations, all within the state of Colorado, with a total of 93 individual PEMA included. Our study was conducted across a significant portion of the range of PEMA (e.g. the conterminous United States), comprising tens of thousands of individual occurrence records. To our surprise, while a Bergmann’s pattern was evident in our data, PEMA body size showed a strong *negative* relationship with mean annual precipitation. This is counter to theoretical and empirical work showing that precipitation predicts intraspecific body size, given that increased precipitation can boost food availability^42^. We also found a pattern of smaller body size in sampled PEMA in the fall. Seasonal changes in body size, including body length, have been found within populations of other rodent species (e.g.^43^), but it is likely that our results indicate age structuring (likely driven by late-season recruitment of juveniles at higher latitudes). More work is needed to understand how adapted niche characteristics of species condition climate-body size relationships.

Our results show that PEMA are shorter but not lighter in more urbanized areas, highlighting the critical need for a standard body size metric in global change studies. This result does not support the Island Rule (the hypothesis that body size increases in urban areas) for smaller mammals, or due to the potential for more available resources for an omnivore. This result is also not consistent with findings by Pergams and Lacy^37^, who found a trend towards longer PEMA over time in the closely related white-footed mouse, *Peromyscus leucopus*, in urban environments in Chicago. Our findings are also inconsistent with the broader assessment of rodent morphological change in response to population density by Pergams and Lawler^44^, who found a slight trend towards larger body size in rodents. It may be that, even at the continental scale at which PEMA occurs, urban heat effects that might drive smaller body size are stronger than the effects of higher available resources and fragmentation in dense urban centers (but see below). As well, changing water and precipitation regimes in urbanized areas compared to surroundings^45^ may also drive body size decreases, given our above climatic results. Finally, it is also possible that selection for smaller HB Length (but not body mass) may be separate from any climate driver. PEMA are considered pests given their propensity to nest in human habitations^46^, and shorter body lengths may simply be a response towards crypsis in novel and climatically more stable microhabitats. Critical to sorting among these hypotheses will be more replications across species with different niche characteristics, as well as standardization of body size metrics across future studies. For example, species with inverse Bergmann’s relationships and with more typical positive relationships between body mass and precipitation may show very different responses to urbanization. Or it may be that smaller sizes are a selective advantage in urban areas in order to avoid human detection that transcend taxonomic diversity.

### Patterns and drivers of body size change over time

Adding decade as a fixed effect and fitting decadal slopes per zone revealed that body mass - but not HB length - is decreasing over time across all zones. Further analysis of the random effects in both models showed a strong negative relationship between the strength and direction of decadal body size change and the overall body size change across zones, for both measures of body size. That is, populations of PEMA at different ends of the body size continuum are responding in different ways to changing environments (populations with larger individuals decreasing, and vice versa for populations with smaller individuals) and this was true regardless of body size metric used. We did not anticipate either of the results for the fixed and random effects, and together they might indicate that body size is homogenizing across time, e.g. mean body sizes across zones becoming more similar in the present than the past. However, we do not find robust evidence that this is the case, and further examination of temporal trends, with full accounting of sampling issues is a next step for quantifying variation change.

While we focused on urbanization and climate change as potential drivers of body size change, we recognize that other footprints of human domination (land conversion besides urbanization, fire frequency, etc.) are missing in our analyses and critical for understanding the results presented here, especially in relation to the finding of contrasting responses of smaller and larger PEMA across time and the possibility of trait homogenization. Our work utilized a unique decadal model of human population density that made it possible to examine direct drivers of trends at a finer grain than is typical, and further efforts to assemble historical land use change layers will permit even more rigorous tests of drivers. Indeed, DiBattista^47^ found genetic variation was differentially impacted by the specific type of human-caused disturbance (i.e., habitat fragmentation, hunting/harvesting, and pollution), as well as the timing and duration of the disturbance. While it may currently be out of reach to test historic range-wide disturbance intensities at a fine enough scale to tease apart those signals, this work at least provides a path towards next-generation analyses that may uncover key generalities across many vertebrate species.

### Issues with combining data streams

We purposefully assembled data from three different sources that have different measurement protocols in order to expose potential systematic biases in how measurements were made. Recognizing and accounting for such biases is imperative for rigorous analysis of shifts in a variety of species traits, especially because disparate data streams must often be harmonized prior to analysis. Measurements on mammalian voucher specimens typically follow a prescribed set of methods^48^ that were codified by giants of 20th century natural history such as C. Hart Merriam and Joseph Grinnell, and passed down to generations of North American mammalogists. These specimen-associated measurements represent a large proportion of existing digital biodiversity data resources for small mammals. NEON (and to a lesser extent, NACSM) followed more census-based methods (including catch-and-release trap surveys) and if anything, we would have assumed that measurements from live-captured individuals would display much lower precision than museum data (e.g.^49^). However, systematic biases in the absolute direction of measurement bias are also detectable: body mass showed no difference across data sources while HB Length was generally much shorter for NEON data than either museum or NACSM data. This discrepancy results from total length measurements made by NEON personnel on live, unanesthetized animals, which are hard to obtain with accuracy due to difficulties in handling and manipulating live individuals. Specifically, NEON measurements on live PEMA are likely to be less accurate and underestimated in almost all cases than for voucher specimens (Thibault, pers. comm.). Conversely, accurate masses for both live and recently dead individuals (collected by weighing individuals within handling bags) should be more commensurate across data types. We note this issue because combining data streams requires care and understanding data collection protocols. If undetected, systematic underestimates of body lengths in NEON data, all collected towards the present, would have generated a stronger, but false, pattern of body size reductions.

### Next steps

Focusing efforts on *Peromyscus maniculatus* was strategic. Because it is widespread and abundant in North American mammal communities, PEMA has a remarkable amount of body size data available over the past century of collecting and has been a model system for understanding life history variation over the past 7 decades. PEMA have multiple generations per year, short generation times, and are also known to be highly adaptable^50^; we thus expected that large-scale environmental change associated with human activities over 70 years could elicit plastic or adaptive trait responses comparable in magnitude to any climate-driven, ecogeographic signatures previously detected. How much of this change related to dispersal-related population replacements, or direct adaptive or plastic change was not addressed here, but museum specimens provide the basis for examining such questions using historical genomic techniques, which are now becoming commonplace^51^. As well, while PEMA are uniquely well sampled, hundreds of other species of North American mammals are also represented by 1000 s to 10000 s of specimen-level body length and body mass measurements compiled by painstaking work of field mammalogists. We reiterate that these datasets require considerable care and quality control when used in broad-scale analyses, but are nevertheless a unique resource to understand range-wide and lineage-wide responses across broad scales, in a time of unprecedented change. An obvious next step is to include many more species utilizing hierarchical modeling frameworks that can borrow strength across many lineages that vary in historic sampling effort, in order to examine body size trends and among-trait variation at the broadest phylogenetic scale and in relation to species-specific evolutionary and ecological differences. While we doubt a single set of trends will emerge when examining a group as diverse as mammals - especially given widely different life histories and niche characteristics, along with variable, and complex interactions between humans and other mammals - we still argue useful predictions about biodiversity response can be made and incorporated into modeling frameworks. Beyond focusing on species response in accordance with ecogeographic rules, it will be critical to parse how anthropogenic habitat modification interacts with (or even counteracts) these more familiar axes of trait variation. For example, body size decreases (like we find here for HB length) may be a more general response of mammals to selection for crypsis or the availability of more stable microclimates in dense human-built environments such as cities.

## Materials and Methods

### Occurrence and trait data aggregation

Body size data for PEMA were obtained from three primary sources. First, we used digitized natural history specimen records aggregated in VertNet (vertnet.org^52^). We obtained standard total body length and mass measurements previously unlocked from the VertNet corpus using the approach of Guralnick *et al*.^14^. We then modified this approach, and developed new parser scripts to obtain additional, detailed body length measurements from specimen records, especially tail-length in this case, applicable for all non-volant mammals (see https://github.com/rafelafrance/traiter). Second, we collected the same body size data from the North America Census of Small Mammals (NACSM^15–23^). NACSM was part of the Rodent Ecology Project at Johns Hopkins University that coordinated small mammal censuses across North America^15^. While most surveyors associated with the study collected measurements from voucher specimens, our data extraction suggests that others were collected from live individuals, thus making this data source a mixture of the two types of measurements. We consulted annual NACSM reports and manually digitized a portion of PEMA records that contained associated head-body length and/or mass measurements. Third, we gathered body size data from the live mammal census records generated by the National Ecological Observatory Network (NEON; https://www.neonscience.org/)^53^. NEON data were extracted using the “neonUtilities” R package^54^. We aggregated all PEMA records that contained body length and/or mass measurements from these sources and harmonized data fields across sources for analysis.

We assembled datasets for two body size traits: body mass and head-body length. For body mass, we used known adult body mass ranges along with juvenile reporting (in VertNet records) to filter out records below 9 grams and reported juveniles, which represent unambiguous juvenile individuals^55^. We derived head-body length (HB Length) by subtracting tail lengths from overall total length^13^. Isolating HB Length is critical because tail length varies substantially in PEMA (and across *Peromyscus*), possibly as an anatomical feature used in locomotion and balance. All measurements were carefully hand-checked to flag obvious outliers, and records with trait measures more than 3 standard deviations from the mean for any measurement (i.e. body mass, tail length, or total length) were also discarded.

Finally, to fill spatial sampling gaps, we manually georeferenced some additional VertNet specimen records for which body size measurements were available. Our georeferencing protocols followed Chapman and Wieczorek^56^ and employed a combination of tools including Google Maps (https://www.google.com/maps) and the MaNIS georeferencing calculator^57^ (http://manisnet.org/gci2.html). Next, we manually georeferenced locations of NACSM census sites and paired the localities with body size data from that resource^13^. We also used verbatim geospatial data associated with NEON census sites for body size data from that resource.

### Processing of specimen trait records for model construction

A final set of preparation steps were needed to create a model-ready dataset. First, we filtered remaining records that could not be georeferenced, that lacked date of collection or otherwise had unusable dates. We also removed those records without day or month reporting, and we removed records with collection dates of “1 January”, since these often represent misreporting of records where only year is known. We removed records that lacked sex reporting as well as those with ambiguous sex assignments such as “undetermined” or “female?”. The vast majority of those filtered records were VertNet records. We then derived two new fields: “season collected” and “decade”. We used month and day of collection to assign records to season categories (e.g. Spring, Summer, Fall, Winter defined by equinox and solstice dates) and we used decade of collection to bin records into decades. We only included records from 1895 onwards and binned into ten year increments. This created 13 decade bins, although the last was from only 2015–2019. For temporal analyses, we further filtered records as described below. Finally, to account for the possible effects of age class and spatially variable population demographic processes, we created alternate versions of our dataset with all juveniles labeled as such in the Darwin Core field “lifeStage” filtered from the analyses. This “no juvenile” dataset was used in analyses below. We also provide supplementary analyses with marked juveniles not excluded, since this increases sample size for analyses, and we do not expect biases in juvenile number over time or space based on collecting methods.

### Pairing climate and population density information with records

Following aggregation from all data sources, we obtained paired historical climate data for georeferenced body size records using ClimateNA v6.0^58^. ClimateNA is a reference tool consisting of past and present climate grids for mainland North America interpolated from long-term weather stations using the method of Mitchell and Jones^59^. We used geocoordinates of all body size occurrences as input into ClimateNA and augmented these with verbatim elevation values for records whenever available; the latter values are used for refining climate estimates within grid cells^58^. Default settings were used for all ClimateNA extractions. We extracted all climate variables for each body size record and later parsed mean annual temperature (MAT) and mean annual precipitation (MAP) of the year of collection for use in statistical analysis. Given the short gestation time (22–30 days) and average life-span (less than one year in the wild), using current year of collection for temperature and precipitation values is warranted.

We assigned human population density estimates to each record using human population density data for the USA from Fang and Jawitz^60^, who provided decadal human population density from 1790 to 2010. This high spatial resolution (1 km by 1 km) modeled human population density dataset serves as a proxy of urbanization and human disturbance. It is also unique in providing temporal depth, which is critical for our use here. Beyond the key temporal resolution provided by this dataset, we prefer population density over impervious land coverage as a measure of urbanization for two reasons. Urbanization is mainly driven by population and is a proxy for many other biotic and abiotic changes. As well, population density ranges from over multiple orders of magnitude while impervious land coverage has an upper limit of 1. Therefore, population density may quantify urbanization and disturbance more effectively. We note that because such data over time is only available in the USA, this step excludes records of PEMA from Canada and Mexico. Our analysis therefore is constrained to the conterminous USA. In order to assign a human population density record to each record, we used an R^61^ script that indexed the decade collected and spatial location of the specimen record to the correct human population density pixel in a stack of layers. We also assembled an estimate of human population density for each specimen record at 1 km, 4 km, and 10 km via pixel aggregation. All values for human population density were logged before use in models given that values can range over orders of magnitude.

### Relationship between head-body length and body mass

We determined the relationship between head-body length and body mass using a simple univariate linear regression where log(HB Length) predicts log(body mass). We performed this analysis and examined variance explained before treating these two variables separately in analyses, since very strong fits would imply co-linearity. More detailed analyses using sex or season covariates are likely to be informative regarding the details of these allometric patterns, but were not done here, because the key prediction we are testing is that the relationship between the two key variables used in the rest of the analyses are relatively weak.

### Defining spatial regions and replicates

We binned body size records into ecoregional categories to account for broad climatic variation across the range of PEMA and associated ecological differences among populations. We developed two binning approaches, used for separate spatial and temporal analyses described below. First, we used Level I ecoregions as defined by the United States Environmental Protection Agency (https://www.epa.gov/eco-research/ecoregions) but divided several ecoregions more finely given the major climate and latitudinal range that some ecoregions encompass. Specifically, we split 3 of the Level 1 ecoregions (‘Great Plains’, ‘Northwestern Forested Mountains’, and ‘North American Deserts’) at 42 degrees latitude and re-designated the resulting ecoregions (‘Northern and Southern Great Plains’, ‘Northern and Southern Cordilleras’, and ‘Northern and Southern Desserts’, respectively; Fig. S1). We then extracted ecoregional membership for PEMA occurrences using the over function in sp^62^ in R.

In order to aggregate PEMA body size records into spatiotemporal replicates at sub-ecoregional scales, we developed a second spatial binning approach employing 200 × 200 km grid cells. The goal of the binning approach was to identify those areas where sampling has been most dense over time, and to use these spatial areas in random effect models as replicates to understand different dynamics of change in temperature, and human population density. To optimize the number of spatial replicates, we developed an R script that sampled individual grid cells at random across the contiguous U.S. and retained cells if they met the following criteria: 1) At least 75 total records; 2) At least 4 decades represented and; 3) At least 10 records per decade. To optimize the sample sizes within grid cells, we resampled cells at random for up to 200,000 iterations, replacing existing cells with new cells if they were overlapping and contained higher total numbers of body size records. Figure 1 shows an example zonation using all data records.

Because the number of records per decade was part of our optimization criteria, we filtered our original full dataset to create temporal datasets that only included records collected since 1945. While this filters records collected in the early part of the 20th century, those records are much sparser than records during the second half, especially given the inclusion of NACSM data that begins in 1948 and provides strong spatial coverage. We show just how sparse data are in Fig. 2 that documents trends in number of records separately for each data source we used.

We provide a summary (Tables 1, S1, S2, Supplementary Results) of the 12 final datasets, covering 2 different key body size traits, with and without labelled juveniles, with and without NEON as a source, and across spatial and temporal filtering, that were used in this analysis. That supplementary material provides information on the total number of records included, and number of zones, for the spatial and temporal datasets.

### Spatial and temporal analyses of body size drivers

#### Spatial analyses

Our first models examine only spatial predictors of PEMA body size and therefore assess Bergmann’s Rule as it is typically conceptualized. We focus separately here and below on body mass and HB Length as response variables. As a multitude of factors may influence changes in PEMA body size, we ran a suite of linear mixed-effects models, using the R package *lme4*^63^, in order to identify what factors influence spatial variation in body mass and HB Length. We ran a series of 29 candidate models, with the global model consisting of five fixed effects: Mean Annual Precipitation (MAP), Mean Annual Temperature (MAT), sex, season collected (coded as Spring, Summer, Fall and Winter), and human population density. We also included two random intercepts, ecoregion and data source (VertNet, NACSM, NEON). The data source covariate is used to assure that there is not a systematic bias in how measurements were taken across the different data resources we assembled. We did this because measurements from live-trapped individuals can be less precise and harder to obtain than from euthanized voucher specimens (e.g.^49^). In all cases, we mean-centered and standardized continuous predictors, and transformed categorical predictors to factors. In addition, we checked model residuals and found no evidence of spatial autocorrelation. In reporting model results, we provide summary statistics needed for interpretation, including model p-values, but emphasize those less compared to model support values. Based on our results, all HB Length models of spatial variation were run a second time but removing NEON data. We also determined the marginal and conditions R^2^s for the best models as determined via model selection.

#### Analyses with decadal covariates

To assess temporal body size trends, we ran a series of 44 candidate models with six fixed factors in the global model, including MAP, MAT, sex, decade, season, and population density. In these models, we also included a random slope of decade nested within zone. As before, these models were separately run for both body mass and HB Length. NEON data were excluded given bias in HB Length and to avoid unaccounted decade by source interactions given the different sampling approach. All sets of spatial and temporal models were run with and without juvenile PEMA. Models were fit using the R package *lme4*. Model fits were assessed and ranked with AICc using the R package *MuMIn*^64^. We examine model diagnostics as above and, in these analyses, particularly examined random effect results to determine correlations between slope and intercept of the random terms since this informs whether larger or smaller PEMA as stratified by zone show similar or different trends in body size change over time. For comparison with the random effects models, we also performed exploratory analyses where we fit body size by decade independently for each zone.

## Supplementary information

Supplementary information.

## Data Availability

All data and code is available on Dryad (10.5061/dryad.8w9ghx3j7). Many of the datasets used here are cleaned and processed from existing repositories. The exception is digitized data from NACSM, which we will provide in two ways. The first is consolidated with key.csv files used in analyses and the second as separate files of just NACSM data for download and sharing, since these are newly digital data.
