# Supplementary material for: Body size trends in response to climate and urbanization in the widespread North American deer mouse, Peromyscus maniculatus
Source: Sci Rep. 2020 Jun 1;10:8882. doi: 10.1038/s41598-020-65755-x (PMC7264193; doi:10.1038/s41598-020-65755-x)
Supplement: Supplementary file 1 — Supplementary information. [file 41598_2020_65755_MOESM1_ESM.pdf]

Supplementary Material

Body size trends in response to climate and urbanization in the widespread North American deer mouse, *Peromyscus maniculatus*

Robert Guralnick[1*], Maggie M. Hantak[1*], Daijiang Li[1], Bryan S. McLean[1,2]

1. Florida Museum of Natural History, University of Florida, Gainesville, FL 32611
2. Department of Biology, University of North Carolina Greensboro, Greensboro, NC 27402

*These authors contributed equally to this work
Correspondence: Robert Guralnick, E-mail: robgur@gmail.com

This document includes
Supplementary Tables S1-S2
Supplementary Figures S1-S9
Supplementary Results

SUPPLEMENTAL TABLES

Table S1. Full model examples to test for (A) variation across space in body mass and HB Length, and (B) temporal body mass and HB Length trends.

(A) Models of Spatial Variation

Body Mass

pema_bm_nn_spat1 <- lmer(body_mass ~ MAP + MAT + sex + season + pop_10km2_log10 + (1 | ecoregion3) + (1 | source), data=pema_nodecade_bodymass_NN2, control = lmerControl(optimizer="Nelder_Mead"))

HB Length

pema_bl_nn_spat1 <- lmer(HB.Length ~ MAP + MAT + sex + season + pop_10km2_log10 + (1 | ecoregion3) + (1 | source), data=pema_nodecade_bodylength_NN2, control = lmerControl(optimizer="Nelder_Mead"))

(B) Model including decadal covariates

Body Mass

pema_bm_dec1 <- lmer(body_mass ~ MAP + MAT + sex + decade2 + season + pop_10km2_log10 + (1 + decade2 | zone), data=pema_dec_BM_no_NEON2, control = lmerControl(optimizer="Nelder_Mead"))

HB Length

pema_bl_dec1 <- lmer(HB.Length ~ MAP + MAT + sex + season + decade2 + pop_10km2_log10 + (1+decade2 | zone), data=pema_dec_HBL_noNEON2, control = lmerControl(optimizer="Nelder_Mead"))

Table S2. Highest-ranking spatial (A-B) and temporal (C-D) model predictors for body mass and HB Length relationships. Results are based on datasets that include juveniles, but do not include NEON as a source. Direction (all negative) of continuous predictor estimates are provided. All predictors in each model were highly significant. The ΔAICc describes the difference in AICc scores between the first and second ranked models.

**Top Model**	**N**	***k***	**AICc**	**AICcWt**	**ΔAICc**	**Marginal R^2**	**Conditional R^2**
**(A) Spatial: Body Mass**							
MAT (-) + MAP (-) + Season + Sex	19644	10	110561.6	0.914	4.73	0.045	0.121
**(B) Spatial: HB Length**							
MAT (-) + MAP (-) + Season + Sex + Population Density (-)	27646	11	192409.8	1	24.62	0.035	0.066
**(C) Temporal: Body Mass**							
MAT (-) + MAP (-) + Season + Sex + Decade (-)	11187	12	62123.8	0.653	1.49	0.078	0.201
**(D) Temporal: HB Length**							
MAT (-) + MAP (-) + Season + Sex + Decade (-)	19650	12	136360.4	0.528	0.53	0.036	0.135

Table S3. Highest-ranking spatial model predictors for body mass and HB Length relationships for datasets including NEON as a source (A-B) without juvenile PEMA and (C-D) with juveniles. Direction (all negative) of continuous predictor estimates are provided. All predictors in each model were highly significant. The ΔAICc describes the difference in AICc scores between the first and second ranked models.

**Top Model**	**N**	***k***	**AICc**	**AICcWt**	**ΔAICc**	**Marginal R^2**	**Conditional R^2**
**(A) Spatial: Body Mass**							
MAT (-) + MAP (-) + Season + Sex	22318	10	124704.7	0.655	1.28	0.048	0.131
**(B) Spatial: HB Length**							
MAT (-) + MAP (-)+ Season + Sex + Population Density (-)	27460	11	190870.2	1	19.41	0.021	0.435
**(C) Spatial: Body Mass**							
MAT (-) + MAP (-) + Season + Sex	22744	10	127257.4	0.802	2.80	0.046	0.128
**(D) Spatial: HB Length**							
MAT (-) + MAP (-) + Season + Sex + Population Density (-)	27914	11	194339.7	1	25.05	0.022	0.416

SUPPLEMENTARY FIGURES

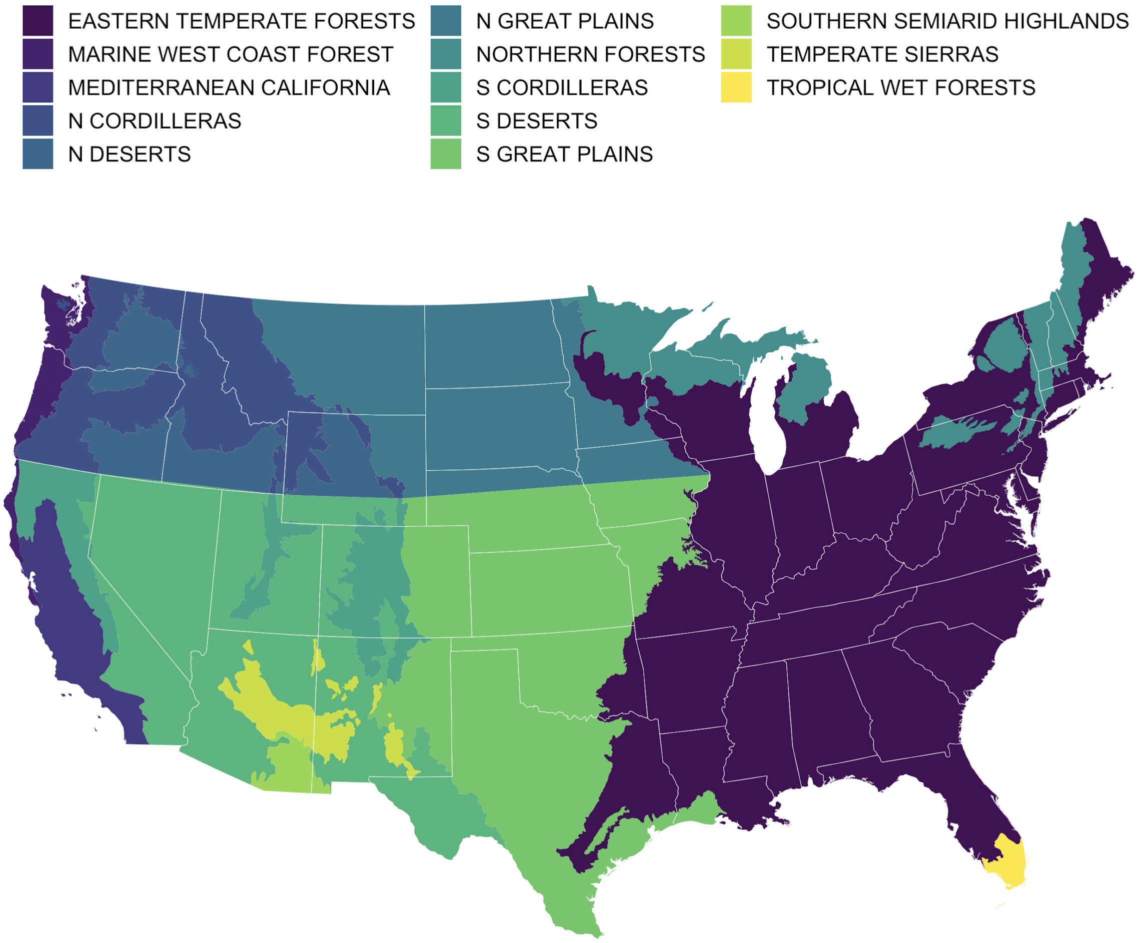

Fig. S1. Ecoregional designations associated with figure 1 in the main document. This map was created with R version 3.6.2 (https://www.r-project.org/).

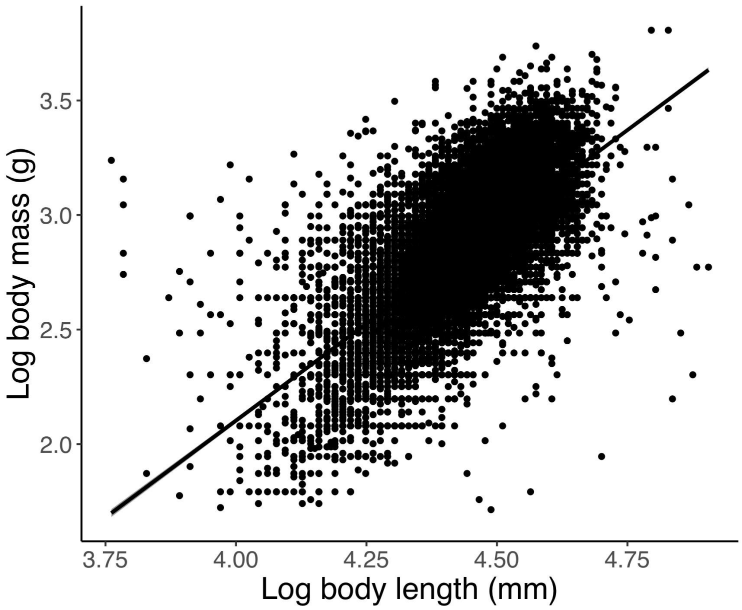

Fig. S2. Scatter plot of the relationship between log(body mass) and log(HB Length).

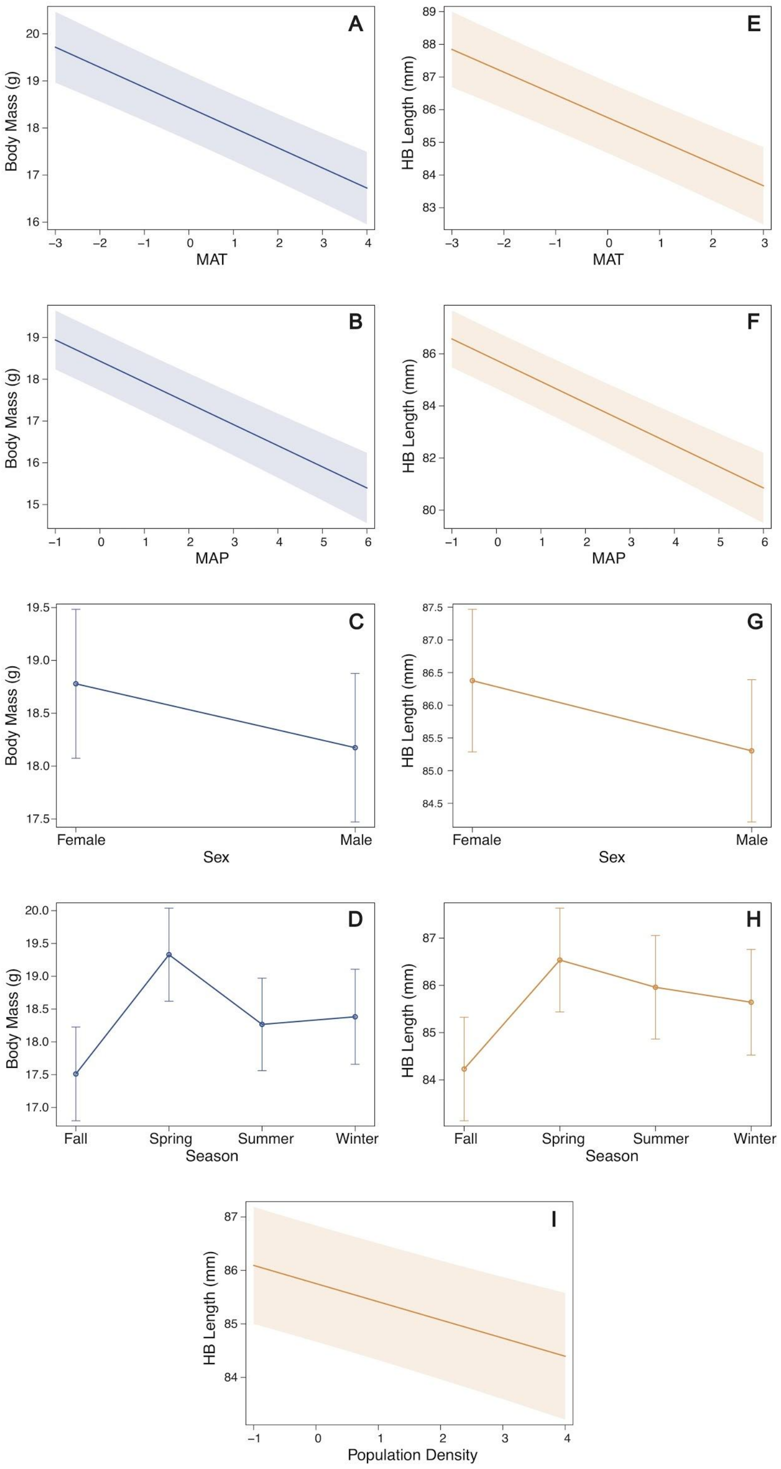

Fig. S3. Top spatial model fixed effect plots demonstrating the effects of MAT, MAP, sex, season, and population density on PEMA (including juveniles, without NEON as a source) body mass (a-d) and HB Length (e-i). 95% confidence intervals are included in each plot.

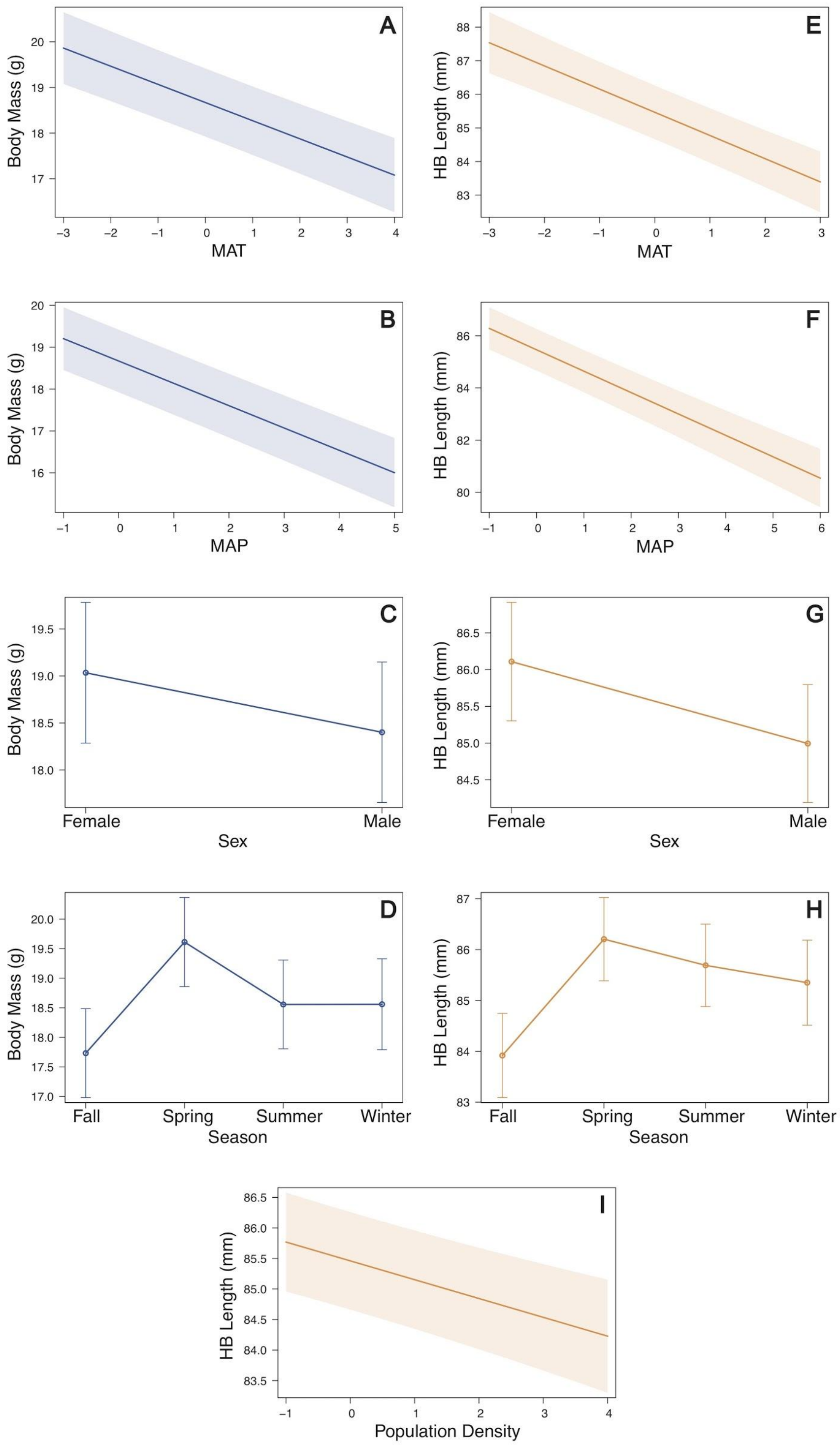

Fig. S4. Top spatial model fixed effect plots demonstrating the effects of MAT, MAP, sex, season, and population density on PEMA (without juveniles and with NEON as a source) body mass (a-d) and HB Length (e-i). 95% confidence intervals are included in each plot.

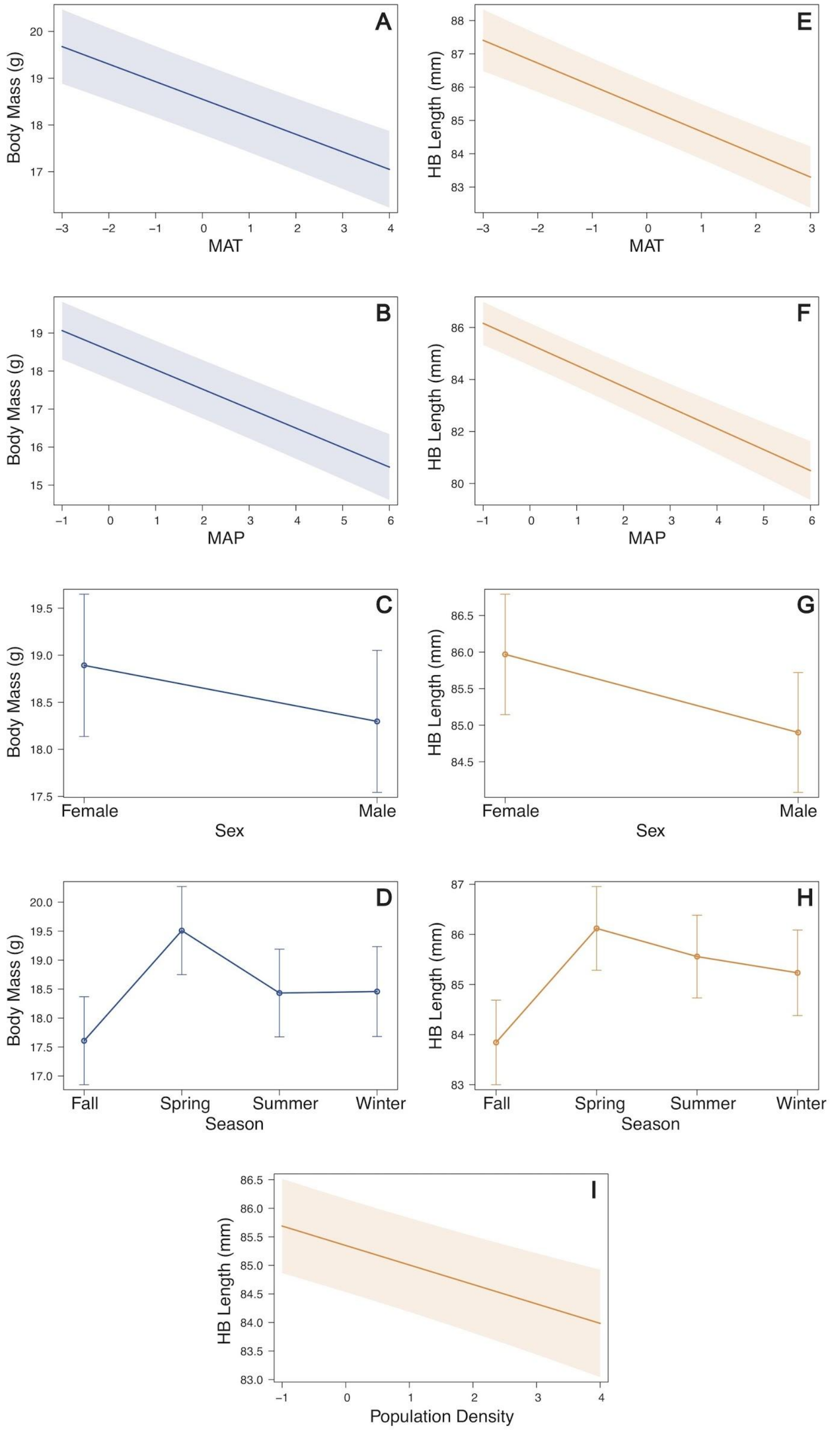

Fig. S5. Top spatial model fixed effect plots demonstrating the effects of MAT, MAP, sex, season, and population density on PEMA (including juveniles and NEON as a source) body mass (a-d) and HB Length (e-i). 95% confidence intervals are included in each plot.

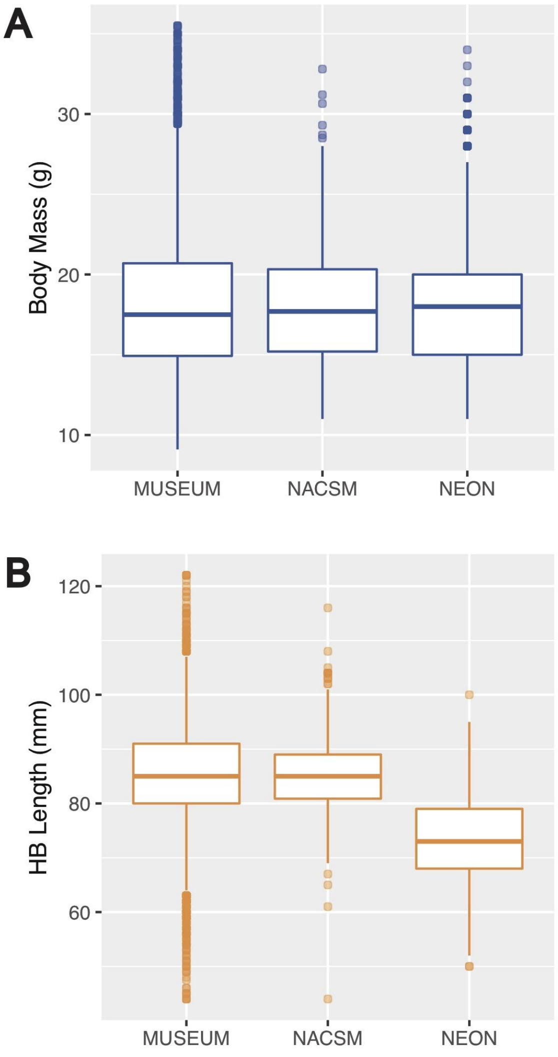

Fig. S6. Boxplots of the random effect of source in spatial models for (A) body mass and (B) HB Length of PEMA (including juveniles). The thick, horizontal line of each box represents the median body size estimate, boxes indicate the interquartile range, and whiskers extend to the largest and smallest body size estimates.

A. Body Mass

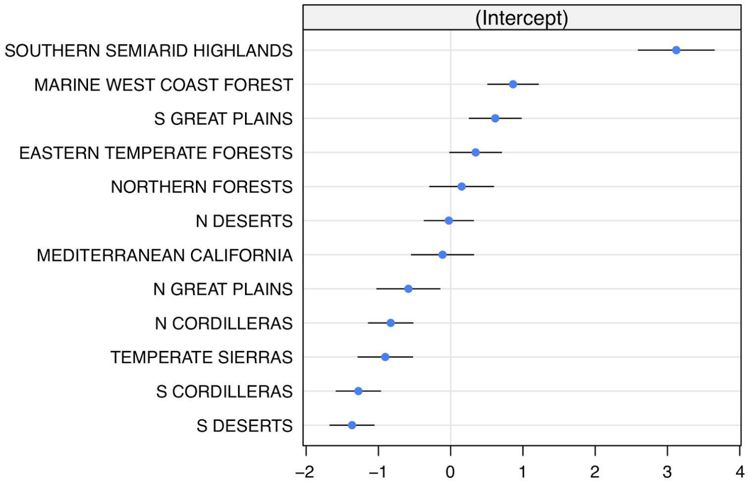

B. HB Length

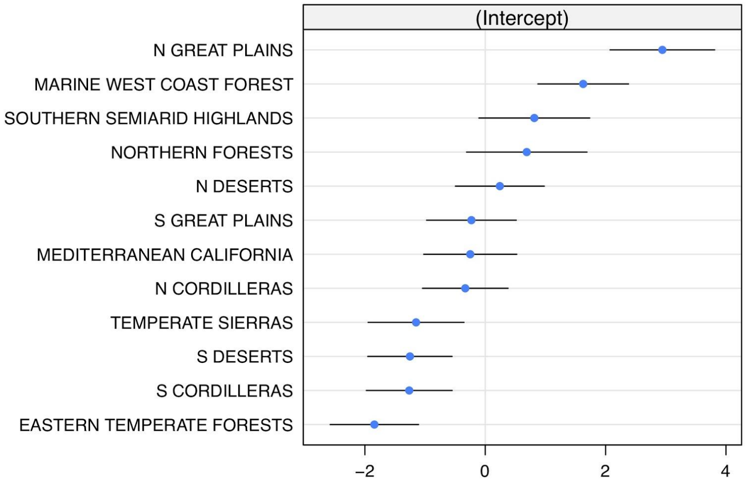

Fig. S7. Caterpillar plots of the random effect of ecoregion in spatial models for (A) body mass and (B) HB Length of PEMA (excluding juveniles and NEON as a source).

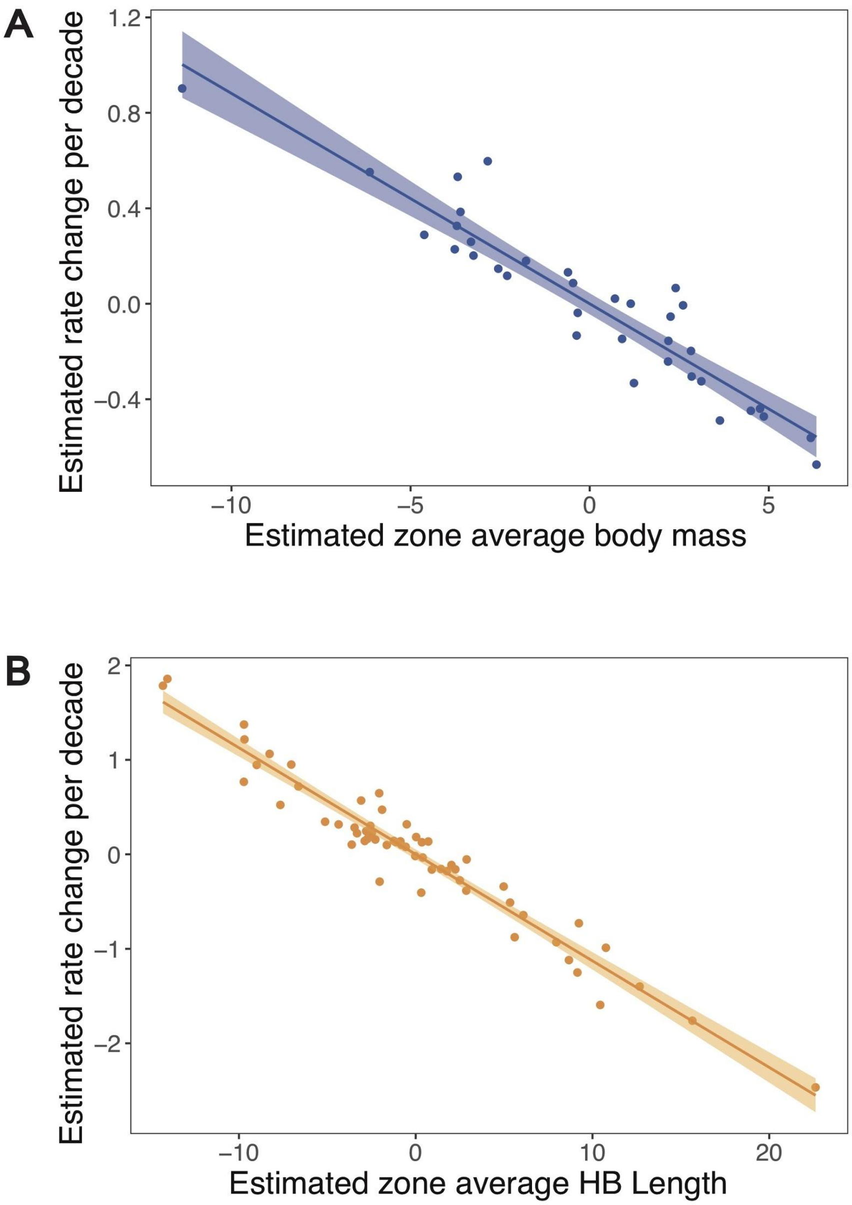

Fig. S8. Scatter plot of the random intercepts of PEMA (A) body mass and (B) HB length against the random slope of decade from the top-ranked temporal models with juveniles.

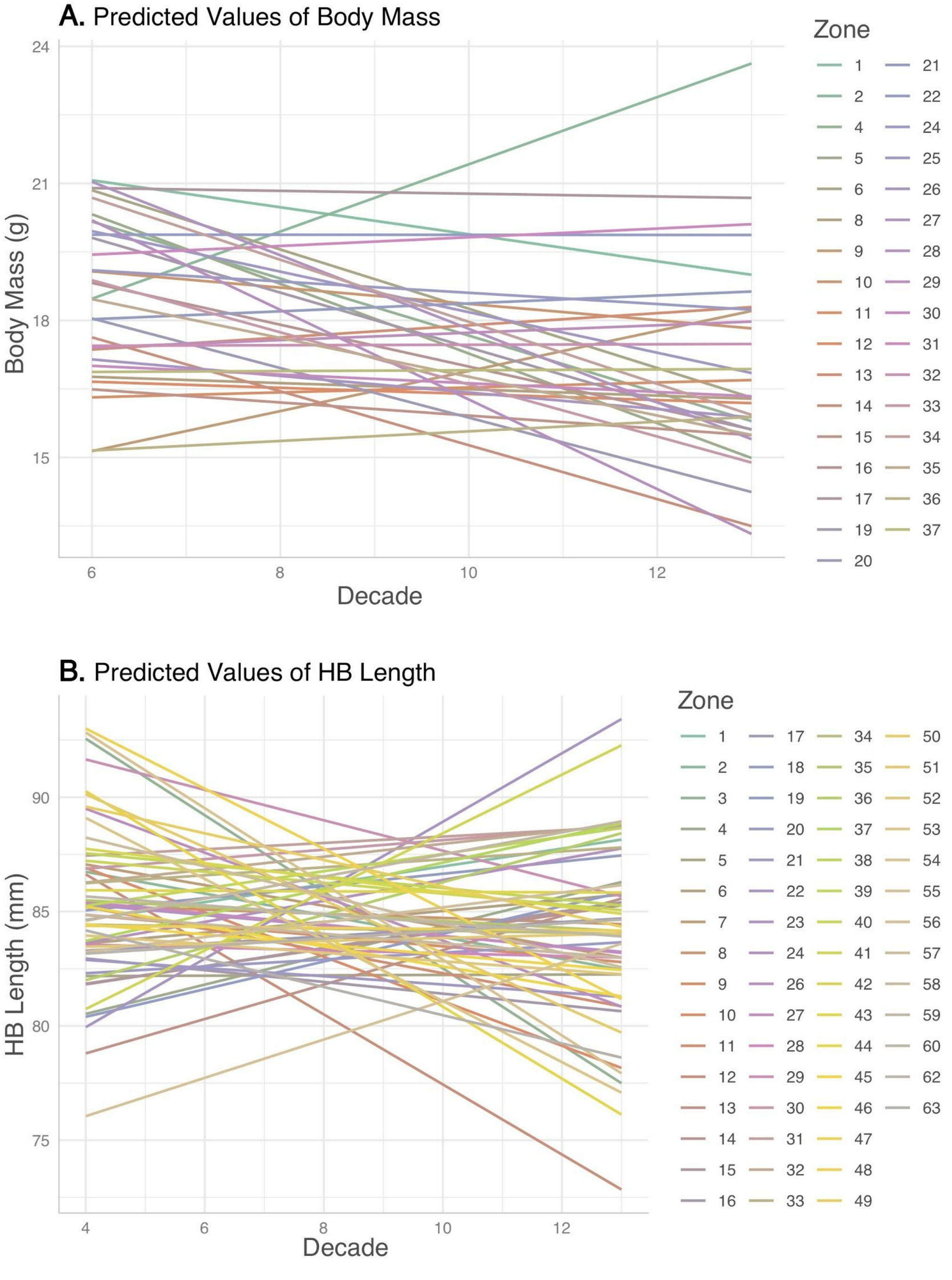

Fig. S9. Top decadal covariate model trends of PEMA (A) body mass and (B) HB Length across decades within each zone.

SUPPLEMENTARY RESULTS

Spatial-only models (including juveniles): PEMA body mass is negatively correlated with increasing MAT (β = -0.43, *SE* = 0.04, $p < 0.001$; Fig. S3a) and MAP (β = -0.51, *SE* = 0.04, $p < 0.001$; Fig. S3b), females are larger than males (β = -0.61, *SE* = 0.06, $p < 0.001$; Fig. S3c), and PEMA body mass is reduced in the fall compared to other seasons (fall-spring β = 1.82, *SE* = 0.10, $p < 0.001$; fall-summer β = 0.75, *SE* = 0.09, $p < 0.001$; fall-winter β = 0.87, *SE* = 0.12, $p < 0.001$; Fig. S3d). HB Length is negatively associated with MAT (β = -0.70, *SE* = 0.07, $p < 0.001$; Fig. S3e) and MAP (β = -0.81, *SE* = 0.07, $p < 0.001$; Fig. S3f). Females display a longer HB Length than males (β = -1.07, *SE* = 0.10, $p < 0.001$; Fig. S3g). PEMA HB Length is shorter in the fall compared to other seasons (fall-spring β = 2.31, *SE* = 0.17, $p < 0.001$; fall-summer β = 1.73, *SE* = 0.15, $p < 0.001$; fall-winter β = 1.41, *SE* = 0.18, $p < 0.001$; Fig. S3h). PEMA HB Length decreased with increasing population density (β = -0.34, *SE* = 0.06, $p < 0.001$; Fig. S3i).

Spatial-only models (including NEON as a source): PEMA body mass is negatively correlated with increasing MAT (β = -0.40, *SE* = 0.04, $p < 0.001$; Fig. S4a) and MAP (β = -0.53, *SE* = 0.04, $p < 0.001$; Fig. S4b), females are larger than males (β = -0.63, *SE* = 0.06, $p < 0.001$; Fig. S4c), and PEMA body mass is reduced in the fall compared to other seasons (fall-spring β = 1.88, *SE* = 0.09, $p < 0.001$; fall-summer β = 0.82, *SE* = 0.08, $p < 0.001$; fall-winter β = 0.83, *SE* = 0.12, $p < 0.001$; Fig. S4d). HB Length is negatively associated with MAT (β = -0.69, *SE* = 0.07, $p < 0.001$; Fig. S4e) and MAP (β = -0.82, *SE* = 0.07, $p < 0.001$; Fig. S4f). Females display a longer HB Length than males (β = -1.12, *SE* = 0.10, $p < 0.001$; Fig. S4g). PEMA HB Length is shorter in the fall compared to other seasons (fall-spring β = 2.29, *SE* = 0.17, $p < 0.001$; fall-summer β = 1.77, *SE* = 0.18, $p < 0.001$; fall-winter β = 1.43, *SE* = 0.18, $p < 0.001$; Fig. S4h). PEMA HB Length decreased with increasing population density (β = -0.31, *SE* = 0.06, $p < 0.001$; Fig. S4i).

Spatial-only models (including juveniles and NEON as a source): PEMA body mass is negatively correlated with increasing MAT (β = -0.38, *SE* = 0.04, $p < 0.001$; Fig. S5a) and MAP (β = -0.51, *SE* = 0.04, $p < 0.001$; Fig. S5b), females are larger than males (β = -0.60, *SE* = 0.06, $p < 0.001$; Fig. S5c), and PEMA body mass is reduced in the fall compared to other seasons (fall-spring β = 1.90, *SE* = 0.09, $p < 0.001$; fall-summer β = 0.82, *SE* = 0.08, $p < 0.001$; fall-winter β = 0.85, *SE* = 0.12, $p < 0.001$; Fig. S5d). HB Length is negatively associated with MAT (β = -0.68, *SE* = 0.07, $p < 0.001$; Fig. S5e) and MAP (β = -0.81, *SE* = 0.07, $p < 0.001$; Fig. S5f). Females display a longer HB Length than males (β = -1.07, *SE* = 0.10, $p < 0.001$; Fig. S5g). PEMA HB Length is shorter in the fall compared to other seasons (fall-spring β = 2.28, *SE* = 0.17, $p < 0.001$; fall-summer β = 1.71, *SE* = 0.15, $p < 0.001$; fall-winter β = 1.39, *SE* = 0.18, $p < 0.001$; Fig. S5h). PEMA HB Length decreased with increasing population density (β = -0.34, *SE* = 0.06, $p < 0.001$; Fig. S5i).

Models including decadal covariates (including juveniles):

We found that PEMA body mass decreases with MAT (β = -0.37, SE = 0.07, $p < 0.001$) and MAP (β = -1.04, SE = 0.08, $p < 0.001$), females display a larger body mass relative to males (β = -0.58, SE = 0.08, $p < 0.001$). Further, PEMA consistently have a smaller body mass in the fall season (fall-spring β = 1.85, SE = 0.14, $p < 0.001$; fall-summer β = 0.70, SE = 0.13, $p < 0.001$; fall-winter β = 1.86, SE = 0.18, $p < 0.001$). We found that PEMA body mass has, overall, decreased over time (β = -0.21, SE = 0.08, p = 0.010 for decade covariate). PEMA HB Length decreases with increasing MAT (β = -0.73, SE = 0.11, $p < 0.001$) and MAP (β = -0.91, SE = 0.11, $p < 0.001$), are shorter in the fall compared to other seasons (fall-spring β = 2.74, SE = 0.21, $p < 0.001$; fall-summer β = 2.16, SE = 0.20, $p < 0.001$; fall-winter β = 2.29, SE = 0.23, $p <$ 0.001), and females are longer (β = -1.02, SE = 0.12, $p < 0.001$) than males. PEMA HB Length has decreased over time (β = -0.35, SE = 0.12, p = 0.004 for decade covariate).
